# The Role of the Immune Phenotype in Tumor Progression and Prognosis of Patients with Mycosis Fungoides: A Quantitative Immunohistology Whole Slide Approach

**DOI:** 10.3390/cells11223570

**Published:** 2022-11-11

**Authors:** Natallia Aulasevich, Maximilian Haist, Sebastian Försch, Beate Weidenthaler-Barth, Volker Mailänder

**Affiliations:** 1 Department of Dermatology, University Medical Center of the Johannes Gutenberg University Mainz, 55131 Mainz, Germany; 2 Institute of Pathology, University Medical Center of the Johannes Gutenberg University Mainz, 55131 Mainz, Germany; 3 Max Planck Institute for Polymer Research, 55128 Mainz, Germany

**Keywords:** mycosis fungoides, immune phenotype, immunohistochemistry, tumor microenvironment, CD30, prognosis, tumor heterogeneity

## Abstract

Background and objectives: Mycosis fungoides (MF) is the most common type of cutaneous T-cell lymphomas, characterized by mature, skin-tropic CD4+ T-helper cells. In order to study the immune tumor microenvironment in MF patients, we performed immunohistochemical stains on MF biopsies, digitized whole-slide tissue sections, and performed quantitative analysis of the different immune cell subsets to correlate tissue parameters with the clinical data of patients, such as progression-free survival or overall survival. Patients and methods: Overall, 35 patients who were treated between 2009 and 2019 and for whom one or more paraffin tissue blocks were available have been included in the present study (58 tissue specimens in total). Conventional immunohistochemistry stains for CD3, CD4, CD8, CD20 and CD30 were used for the analysis of the immune phenotype, and quantitative analysis was performed using QuPath as a quantitative digital pathology tool for bioimage analysis of whole slides. Results: Analysis of tissue parameters for prognostic significance revealed that patients with a stronger infiltration by CD8+ lymphocytes within the tumor cell compartment had a higher risk of disease progression (*p* = 0.031) and showed a shorter progress-free survival (*p* = 0.038). Furthermore, a significant association of the percentage of CD30+ cells (median: 7.8%) with the risk of disease progression (*p* = 0.023) and progression-free survival (*p* = 0.023) was found. In relation to the clinical features of our patient cohort, a higher risk of disease progression (*p* = 0.015) and a shorter progression-free survival (*p* = 0.032) for older patients (>61 years) were observed. Conclusions: Our results demonstrated the prognostic relevance of large-cell transformation in mycosis fungoides and its strong association with the presence of CD30+ lymphocytes. Unlike previous reports, our study suggests an adverse prognostic role for CD8+ T cells in patients with mycosis fungoides. Moreover, our data indicate that the immune phenotype within the tumor microenvironment shows strong temporal heterogeneity and is altered in the course of tumor progression.

## 1. Introduction:

Cutaneous T-cell lymphomas (CTCL) are a rare and heterogeneous group of T-cell malignancies that occur primarily in the skin [1]. Mycosis fungoides (MF) is the most common type of CTCL and originates from mature, skin-tropic CD4+ T helper cells [2]. Together with Sézary syndrome, which is the leukemic variant of CTCL, MF accounts for 50–60% of all CTCL [3,4]. 

Early-stage MF (IA-IIA) is clinically characterized by patches or plaque-like lesions, which can resemble benign inflammatory skin conditions, such as eczema and psoriasis [5]. Histological features of these early lesions show a substantial overlap with these inflammatory skin disorders, including band-like dermal infiltrates of T-lymphocytes and an intraepidermal collection of lymphocytes, termed Pautrier’s microabscesses, which are particularly common in plaque-stage disease. In these early stages, lymphocytes are typically of small or medium size and show a T-helper memory phenotype (CD3+, CD4+, CD8, CD45R0+). Due to the substantial overlap with inflammatory skin disorders, the diagnosis of early MF still represents a difficult challenge for dermatopathology. The correlation to the clinical findings is of utmost importance and can be complemented by molecular and immunopathological criteria [3]. 

Although patients with early patch or plaque stage MF generally show an indolent course of the disease with a 10-year disease-specific survival rate over 80%, some patients progress to the late tumor stage (IIB-IIIB), which may present with nodal or extracutaneous dissemination (stage IV) [2]. Eventually, a third of patients may show a progression of MF or initially present with an advanced stage of MF, which is associated with a reduced 10-year disease-specific survival of <20% [1,2]. Histopathological features of this advanced MF tumor stage are a dense, diffuse lymphocytic infiltrate of large, pleomorphic, anaplastic or immunoblastic cells, which may present with an aberrant phenotype with a loss of T-cell antigens, and some cases may also present with an CD8+ or TCRγδ+ phenotype [6]. 

Currently, the treatment of MF comprises skin-directed therapies for early-stage disease and systemic therapy for advanced stages of the disease. However, with the exception of hematopoietic stem cell transplantation, there are currently no curative therapies for advanced CTCL, and current systemic treatments mostly only induce short-lived, partial disease control [1,7]. 

In order to allow for an early identification of patients at risk of disease progression or with a worse prognosis who are eligible for systemic treatments, there is a vital need for prognostic markers. The Cutaneous Lymphoma International Consortium identified four independent adverse prognostic markers, namely stage IV disease at initial diagnosis, age > 60 years, large-cell transformation (LCT) and increased LDH. Moreover, it has been suggested that certain histopathological features may indicate a higher risk of disease progression, such as a loss of CD7, increased cerebriform lymphocytes, decreased CD8+ lymphocytes and increased proliferation (Ki67+ lymphocytes) [8,9]. Despite these recent advances in the prognosis of patients with advanced CTCL, to date, biomarker studies using immunohistochemistry (IHC), as well as clinical studies, have not yet provided a comprehensive and specific index predicting the risk of disease progression and an individual patient´s prognosis. The conflicting data of various studies [2,6,10,11,12,13] may be related to the small size of the groups studied, the subjective character of the histopathological interpretation of early MF with poor reproducibility of diagnosis, and the difficulty of distinguishing between the T-cell tumor cells and the reactive T-cell infiltrate in IHC studies, which remains a considerable challenge [1,2]. 

Here, methods of digital pathology have been shown to facilitate an objective and reproducible assessment of MF lesions and help to identify novel prognostic IHC markers for MF patients [1]. In particular, the analysis of the infiltrating cells, or, as determined today, the tumor microenvironment (TME) within MF, may help to predict the risk of disease progression and the prognosis of patients with both early-stage and advanced-stage MF, since tumor-specific immune responses exert an important role in the control of malignant diseases [7]. Notably, IHC analysis allows us to assess the spatial organization of immune cells within the TME, which has been found to be an important prognostic factor in CTCL [1]. Clearly, we cannot distinguish between IHC malignant cells and infiltrating bystander cells. This could only be done by single-cell analysis, e.g., by methods of next-generation sequencing. 

In our investigations, we used IHC stains on patient MF tissue sections, which were available in our department as part of routine diagnosis and follow-up studies for daily patient care. We here show the combination with the digitalization of whole tumor slices. This methodology enables single-cell-based quantitative analyses of the different immune cell subsets within the MF TME in a quantitative and spatial context. In addition to the common lineage markers of CTCL, such as CD3, CD4 and CD8, we have assessed the expression of CD20 and CD30 in a total collection of 58 MF tissue specimens, which have been collected from 35 patients at different time points in the course of MF disease progression. Therefore, we have been able to (1) correlate tissue parameters with the clinical data of our patient cohort, such as progression-free survival (PFS) or overall survival (OS). For some patients, we have collected several tissue samples at different time points in the course of MF progression. This allowed us to (2) evaluate the temporal transformation of the immune phenotype in these patients and correlate microenvironmental changes with the risk of disease progression. 

We speculated that the age of patients at initial diagnosis, the occurrence of large-cell transformation (LCT), a strong expression of CD30 on lymphocytes, and a higher CD4/CD8 ratio might represent adverse prognostic factors, and that patients at higher risk of disease progression might present more often with LCT, folliculotropism and epitheliotropism. 

## 2. Patients and Methods

### 2.1. Patient Cohort

We retrospectively examined 35 patients diagnosed with MF who were treated at the University Medical Center Mainz between 2009 and 2019. Treatments varied depending on the stage of MF and comprised either topical treatment (corticosteroids), phototherapy, chemotherapy (CHOP, gemcitabine), radiotherapy or immunotherapy (monoclonal antibodies). Patients enrolled in blinded randomized controlled trials were excluded due to the uncertainty of therapy assignment.

Data on baseline demographics, tumor specifics (i.e., localization of the tumor, skin manifestation at initial diagnosis, T-cell receptor clonality, tumor stage at initial diagnosis), tumor-specific treatments, survival data and the status of the patient at the time of data lock (30 July 2021) were collected by medical chart review. 

Of the entire cohort of 35 patients, all 35 patients for whom one or more paraffin tissue blocks were available were included in the present study (58 tissue specimens from 35 patients in total). Tissue specimens have been excised at initial diagnosis. For some patients, subsequent tissue specimens have been excised in the course of disease progression (16 of 35 patients). 

For the analysis of the MF phenotype, we performed conventional immunohistochemistry (IHC) stains for CD3, CD4, CD8, CD20 and CD30. All 58 tissue specimens were evaluated with the methods detailed below. Since this approach resulted in more than one tumor sample for some patients, a meaningful reduction of the quantitative data to a single score became necessary in order to allow for a correlation of IHC data with clinical survival data. Therefore, tumor specimens with the highest overall cell count were considered to represent the patient´s individual lymphoma best and were selected for correlation with the patients´ clinical data. We also carried out a separate analysis including only patients for whom two or more subsequent tissue specimens have been available (16 of 35 patients with 38 tissue specimens) to allow for a qualitative assessment of the MF phenotype in the course of disease progression.

### 2.2. Immunohistochemistry Staining

Conventional IHC staining for antigens CD3, CD4, CD8, CD20 and CD30 was carried out according to a standard procedure. In brief, after cutting 2.5 µm thick sections with high precision microtomes, specimens were incubated at 60 °C for one hour and deparaffinized in a descending alcohol series. Pretreatment for IHC was conducted using antigen-demasking buffers specific for the chosen antigen (details are given in Table 1). Subsequently, blocking of the endogenous peroxidase was carried out using a 3% H_2_O_2_ solution. Antibody incubation took place for one hour at 30 °C. Primary antibodies were detected using appropriate secondary antibodies (peroxidase-labeled polymer or anti-rabbit/mouse IG detection system). Tagging was carried out using either 3,3′-diaminobenzidine (DAB) or PermaRed/AP-Auto (substrate/chromogen system). Finally, all specimens were counterstained with haematoxylin using an incubation time of 2 min. After rinsing with tap water, samples were treated with an ascending alcohol series and covered with a coverslip using Eukitt mounting medium. In parallel, we have performed haematoxylin and eosin stains for all tissue specimens. 

### 2.3. Quantitative Analysis

**Figure 1:**
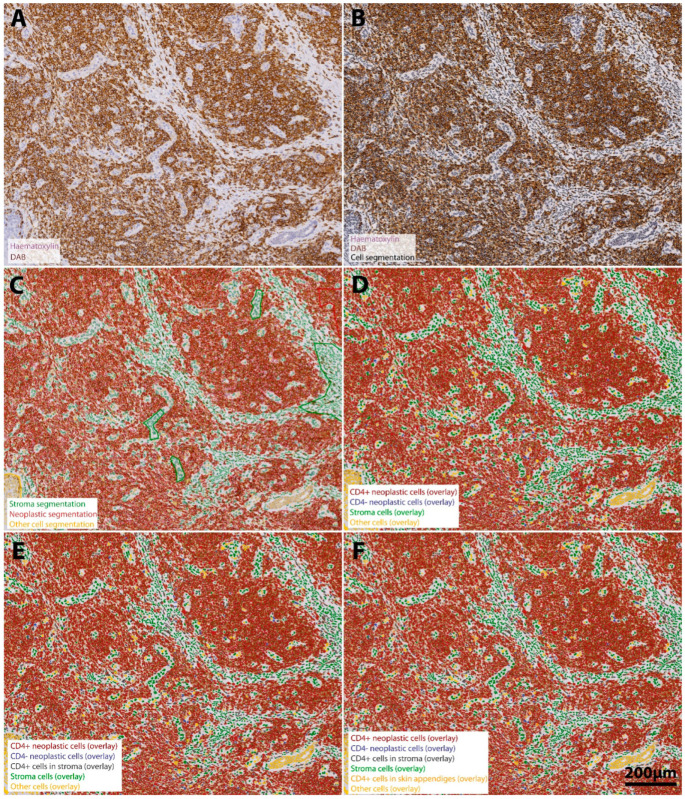
Stepwise procedure for the analysis of the number and spatial context of CD4+ neoplastic lymphocytes in the MF tumor microenvironment. (**A**) Original scanned conventional immunohistochemistry image. A representative region of interest (ROI) (4.6x) extracted from the whole-slide specimen of a patient´s tumor demonstrates intense staining for CD4-positive cells (DAB-channel), which reflects the predominant role of CD4+ lymphocytes in mycosis fungoides. Neoplastic cell infiltrates are predominantly located in the dermal structures and show only minimal epidermal infiltration but a strong folliculotropism. Haematoxylin counterstain (purple) was used for segmentation of the cell nuclei (**B**). Single-cell-based analyses were conducted using the segmented nuclei (black border) as a prerequisite for cell detection in the open-source software QuPath. First, we subclassified cells as “neoplastic lymphocytes” (tumor compartment, red), “stroma cells” (green) (including reactive T cells) or other cell types (comprising epidermal cells and skin appendages, yellow) using QuPath´s machine learning features and user-defined examples (**C**). We further subclassified cells of the three different categories using intensity thresholds in the DAB-channel in a sequential procedure, color-coding CD4+ lymphocytes with a red overlay and CD4− lymphocytes with a blue overlay (**D**). Next, stroma cells were further subclassified into CD4+ reactive lymphocytes (anthracite) and other stroma cells (olive green) (**E**). Finally, we identified CD4+ immune cells infiltrating epidermal and dermal structures (orange overlay) to assess the degree to which lymphocytes may infiltrate the epidermis and skin appendages (**F**). The result of our classification algorithm is shown in panel F. Scale bar in F applies to all images.

Single-cell-based analyses were carried out using the haematoxylin channel for the segmentation of cell nuclei in the open-source whole-slide image analysis software QuPath v0.2.0-m12 [https://qupath.github.io/; [14]]. Segmentation was followed by a stepwise procedure for further subclassification of cells (Figure 1). First, cells were classified as “neoplastic cells (termed tumor)”, “stroma (including reactive T cells)” or “other” (comprising epidermal structures, skin appendages tissues, etc.) using user-defined examples and QuPath’s machine learning features as described previously [15]. Tumor and stromal cells were further subclassified using intensity thresholds in the relevant chromogen channels (DAB). This subclassification comprised the identification of CD3-positive, CD4-positive or CD8-positive lymphocytes, CD20-positive B-cells or CD30-positive lymphocytes either within lymphoma tissue infiltrates or stromal areas. To calculate the distance from each cell classified as an immune cell to the nearest epidermal cells, we used a custom script in QuPath. The assembled quantitative data were correlated with clinical patient survival data, such as PFS or OS. In order to account for the intraindividual lymphoma heterogeneity in the course of disease progression, we did additionally compare the immune cell composition within each tissue specimen of a given patient at different time points of tumor progression and between different localizations of tissue excision.

### 2.4. Spatial Analysis

Spatial analyses were carried out in order to assess the spatial correlation between different immune cell populations, a marker-positive immune cell population and epidermal structures. To this end, we have chosen representative regions of interest from the whole slide specimens in QuPath. Coordinates of cell centroids were used to calculate the distance from immune cell infiltrates to epidermal structures and estimate the direction and strength of the interaction between marker-positive tumor cell populations and epidermal structures. 

### 2.5. Statistical Analysis

Descriptive statistics were used to analyze the baseline characteristics of the study population. PFS was calculated from the start of initial treatment until the date of radiological or clinical disease progression, the last follow-up, or death for any cause. Overall survival (OS) was calculated from the date of initial diagnosis until the date of death or last follow-up. Chi-square test was used to assess the association between categorial variables. The confidence intervals (CI) of 95% of categorical variables were calculated using the Clopper–Pearson method. Comparisons between categorial and continuous variables were performed using a Student’s *t*-test. Moreover, the statistical analysis included the Mann–Whitney test and Spearman’s correlation analysis. These tests were performed for the quantitative analyses of the different immune cell populations to define significant correlations between the amounts of different immune cell populations (two-sided Spearman’s correlation analysis).

As a time-to-event endpoint, this retrospective cohort study used both the PFS and OS, which were estimated using the Kaplan–Meier product-limit method and log-rank statistics in R [16]. The median duration of follow-up was calculated using the reverse Kaplan–Meier method. In all cases, two-tailed *p*-values were calculated and considered significant with values of *p* < 0.05. SPSS (version 27, IBM, Ehningen, Germany), RStudio (Version 1.3.1093) and GraphPad PRISM (Version 5, San Diego, CA, USA) were used for all analyses.

## 3. Results

### 3.1. Discrimination of Malignant from Reactive T Cells in the CTCL Tumor Cell Compartment, Stroma and Other Tissue Elements in Preparation for the Analysis

To allow for a systematic analysis of the whole-slide tissue samples, we first separated the compartment with lymphocytes (in the following referred to as “tumor cell compartment”) from the surrounding stroma and other tissue elements, namely the epidermis, skin appendages, etc. 

H&E-stained sections from all biopsies were reviewed by a board-certified pathologist (B.W.B.) and allowed for the subsequent identification of the presumably lymphocytic cell compartment. As described in the methods section, after recognition of the lymphocyte cell infiltrate compartment by QuPath´s cell segmentation algorithm, samples were separated into tumor, stroma and other dermal compartments, including hair follicles and epidermis, and then subclassified for immune cell marker expression patterns. This preparatory step allowed for the quantification of the tumor and stromal cells in each histology section and to perform all further quantitative analyses separately for the three compartments. 

### 3.2. Expression of T-Cell-Associated Markers CD4, CD8 and CD30 and Associated Findings

We examined tissue samples stained with H&E and conventional IHC for immune-associated markers in a cohort of 35 patients. This clinical data set with extended follow-up (median follow-up: 64 months) allowed us to (i) evaluate the temporal transformation of the immune phenotype in the course of disease progression and (ii) correlate tissue parameters with the survival data of the patients. At initial diagnosis, patients were predominantly of older age (median: 61 years) and presented with a plaque stage (65.7%). 

**Figure 2:**
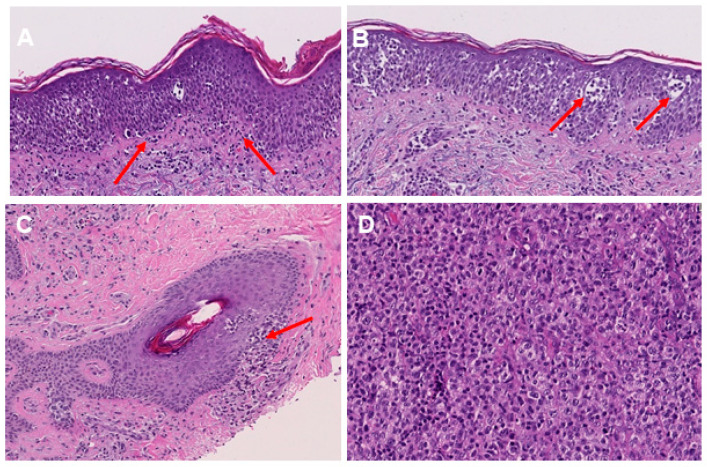
Histological features of MF in H&E staining. Lining up of lymphocytes along the dermo-epidermal junction (indicated by red arrows) (**A**). Lymphocytes within the epidermis, including Pautrier’s microabscesses (as indicated by red arrows) (**B**). Lymphocytic infiltration of hair follicles in plaque stage MF (indicated by red arrows) (**C**). Enlarged and pleomorphic T lymphocytes in the tumor stage of MF (**D**).

We therefore assessed the expression level of lymphocyte markers CD3, CD4 and CD8 in all examined tissue sections (see Table 2 for detailed information on baseline patient characteristics and histological features of the examined tissue samples). We could observe a strong expression of T-lymphocyte markers in all tissue sections. CD4+ lymphocytes were the predominant subtype among all stained tissue sections, which is in accordance with the classical predominance of the helper/inducer phenotype in mycosis fungoides (CD3+, CD4+, CD8−). On the other hand, CD8+ T-cell infiltration was found to be much weaker, although in some tumor specimens, we observed a marked increase in CD8+ T-cells that are presumably mainly infiltrating/bystander cells. Only a more detailed single cell analysis could reveal if they are also malignant, i.e., if they are clonal and would harbor gene modifications that would render them to be malignant or if the CD4+ also acquired CD8+. Notably, the expression of T-lymphocyte markers has not been associated with the overall tumor burden within the examined tissue samples (*p* = 0.237). In accordance with the classical description of MF, we could observe that the spatial distribution of T-lymphocyte subsets met our expectations, as T-lymphocytes in the patch and plaque stages were mainly found lining up along the dermo-epidermal junction zone (Figure 2A) and infiltrating the epidermis to varying degrees (in the following termed as “epitheliotropism”) (Figure 2B). In some cases of plaque stage tissue specimens, we could even observe a beginning infiltration of the skin appendages, termed “adnexotropism” (Figure 2C), whereas in the tumor stage, hair follicles and perspiratory glands have already largely been destructed by the dense, nodular infiltrate of anaplastic lymphocytes, showing a high level of mitotic activity.

Despite the fact that MF is classified as an indolent CTCL, transformations to a more clinically aggressive disease may occur in 10% of cases. Transformed MF may be associated with the appearance of a CD20 component as well as infiltrating cells. Therefore, we have also assessed the expression of CD20 in the examined tissue sections. We could observe that in most of the cases, there was only a very small proportion of CD20+ cells within the investigated samples (<10.0% in 77.4% of cases). On the other hand, a stronger infiltration of CD20+ cells (>10% in 22.6% of cases) has mainly been found in tissue samples from a plaque stage or tumor stage, which might reflect the reactive, inflammatory character of the MF lesions with the CD20+ B cells being attracted by a hitherto unknown chemokine of the primary malignant T cells. 

We further investigated the expression of CD30 in the tissue samples, since CD30 positivity may particularly occur in patients with transformed MF and patients with an aggressive course of the disease [17,18,19]. Overall, our analysis revealed a weak expression of CD30 in the investigated tissue samples (median: 7.8%; 95% CI: 2.2–26.8%). However, it has been found that a strong expression of CD30 was mainly confined to tissue samples excided from patients in the tumor stage of the disease (25% of all tissue samples excided from a tumor stage MF showed a CD30 positivity > 7.8%), whereas a strong CD30 positivity was rarely found in the early MF stages (6.6% of all 45 tissue samples excided from a patch, plaque or parapsoriasis lesion). The morphologic and immunohistochemical features of these cases with high CD30 positivity at an early MF stage were, however, similar to those of other patch/plaque-phase MF cases, and the clinical course of these cases did not differ from that of other cases with early MF and weak CD30 expression. 

In accordance with previous reports, which have shown a close association of tumor stage and the occurrence of large cell transformation (LCT), we could also observe that LCT did mainly occur in tissue samples excided from advanced stage MF (58.3% of all tumor stage tissue samples) (Figure 2D), but was rarely found among early-stage MF patients (6.6% of all 45 tissue samples excided from early-stage MF). Overall, the incidence of LCT in the examined patient cohort was 8.7–29.9%. By contrast, the incidence of folliculotropism—another factor that has earlier been suggested to correlate with the individual patient´s prognosis—was relatively high (37.3%) and was found both in the early stages (26.6%) and advanced stages (41.6%) of MF (*p* = 0.369). 

**Table 2:** Baseline patient’s characteristics and treatment regimens.

Clinicopathological Features	Number (%)
Total number of patients	35
Median age at diagnosis of MF	61.0 years
Gender	
Female	13 (37.2%)
Male	22 (62.8%)
**Primary tumor**	
**Localization**	
- Head/Neck	2 (5.7%)
- Upper limb	10 (28.6%)
- Lower limb	11 (31.4%)
- Trunk	9 (25.7%)
- Genitoanal	3 (8.6%)
Skin manifestations at initial diagnosis *	
- Patch	4 (11.4%)
- Plaque	23 (65.7%)
- Parapsoriasis	1 (2.8%)
- Tumor	7 (20.0%)
**Histology and quantitative data from IHC analysis**	
Large cell transformation	7 (20.0%)
Folliculotropism	13 (37.1%)
Overall tumor load within the examined tissue sections	18.9% (95% CI: 2.6–29.8%)
CD3+ T cells within tumor infiltrates	95.2% (95% CI: 81.7–98.3%)
CD4+ T cells within tumor infiltrates	80.6% (95% CI: 59.85–90.4%)
CD8+ T cells within tumor infiltrates	27.8% (95% CI: 8.5–41.2%)
CD4/CD8 ratio < 2	8/35 (22.9%)
CD30+ T cells within tumor infiltrates	7.8% (95% CI: 2.2–26.8%)
CD20+ cells within the examined tissue sections	6.6% (95% CI: 2.3–25.4%)
Epidermal infiltration by T cells	4.5% (95% CI: 1.5–8.1%)
T-cell receptor clonality	29 (82.9%)
**Follow-up and Treatment**	
**Treatments**	
- Topical treatment	29 (82.9%)
- Phototherapy	20 (57.1%)
- Chemotherapy	8 (22.6%)
- Radiotherapy	10 (28.5%)
- Immunotherapy (i.e., Mogalizumab, Brentuximab, IFN-α)	9 (25.7%)
Progress	12 (34.3%)
Progression to tumor stage in the course of MF	12 (34.3%)
Median progression-free survival after initial diagnosis (95% CI)	215.0 months (37.1–392.9 months)
Median follow-up after initial diagnosis of MF (95% CI)	64.0 months (45.1–82.9 months)
Median overall survival	313 months
Deceased	6 (17.1%)

**Figure 3:**
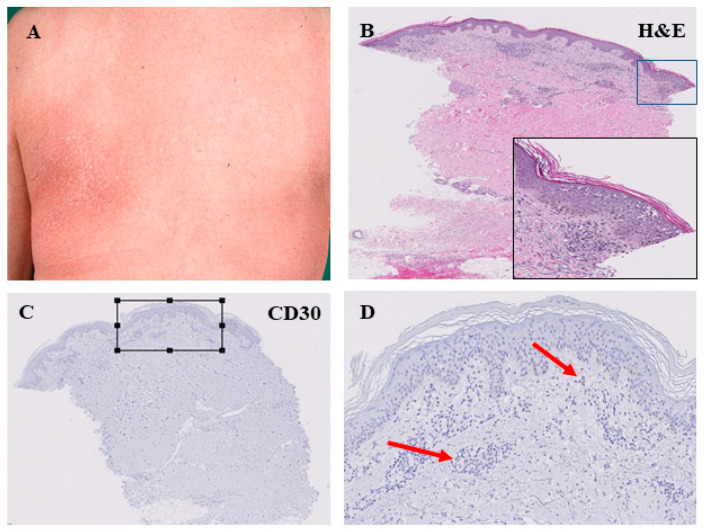
Histopathological features of the patch stage of mycosis fungoides. Large, erythematous lesion in the left subscapular area (**A**). Perivascular infiltration of lymphocytes in the superficial dermis. Lining-up of lymphocytes along the dermo-epidermal junction. Intraepidermal collection of lymphocytes with formation of Pautrier’s microabscesses (**B**). This patch-like stage of MF was associated with low CD30 expression within the lymphocyte infiltrate as indicated by arrows in (**C**,**D**); (**D**) is an enlarged view of the square in (**C**)).

**Figure 4:**
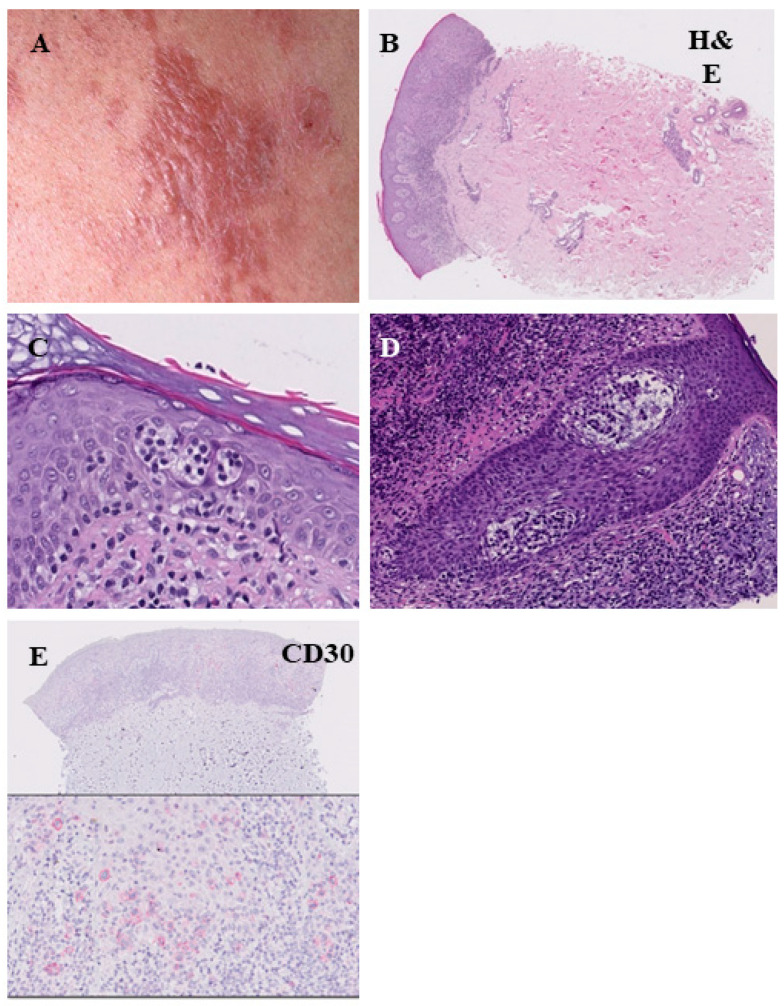
Histopathological features of the plaque stage of mycosis fungoides. Infiltrated reddish-brown lesion with elevation above the surrounding skin (**A**). The infiltrate of lymphocytes is denser and more continuous compared with the patch stage, marked epitheliotropism ((**B**), H&E = hematoxylin-eosin staining). Pautrier’s microabscesses (**C**). Infiltration of lymphocytes within the hair follicles (folliculotropismus) (**D**). Low CD30 expression (**E**).

**Figure 5:**
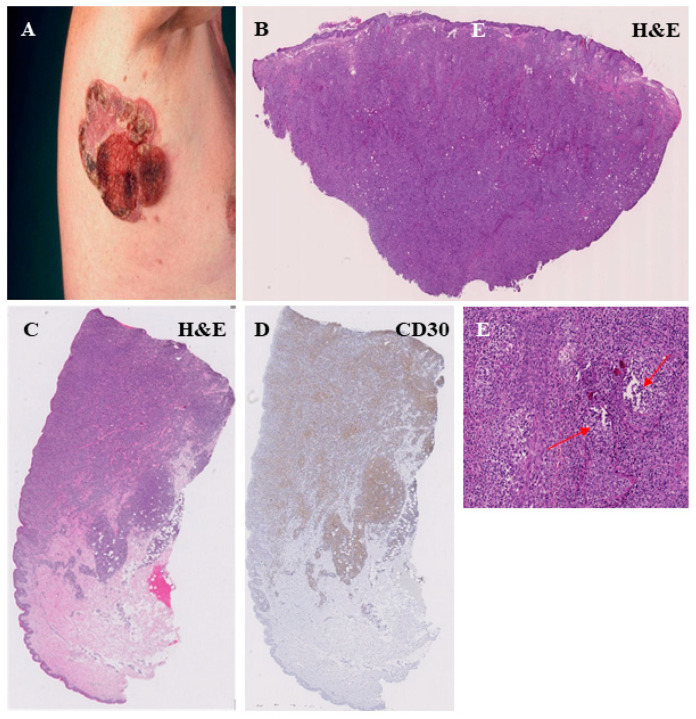
Histopathological features of the MF tumor stage. Clinical representation of a large ulcerated tumor on the right axillary region (**A**). Dense infiltrate of atypic, T lymphocytes with dermal involvement (**B**,**C**) and a strong CD30 expression of the lymphocytes (**D**). The MF tumor stage is often associated with a strong folliculotropism, characterized by a strong infiltration of hair follicles by lymphocytes and a disruption of the follicular architecture (red arrow) (**E**).

* See Figure 3, Figure 4 and Figure 5.

### 3.3. Clinical Correlates of the MF Immune Phenotype

**Figure 6:**
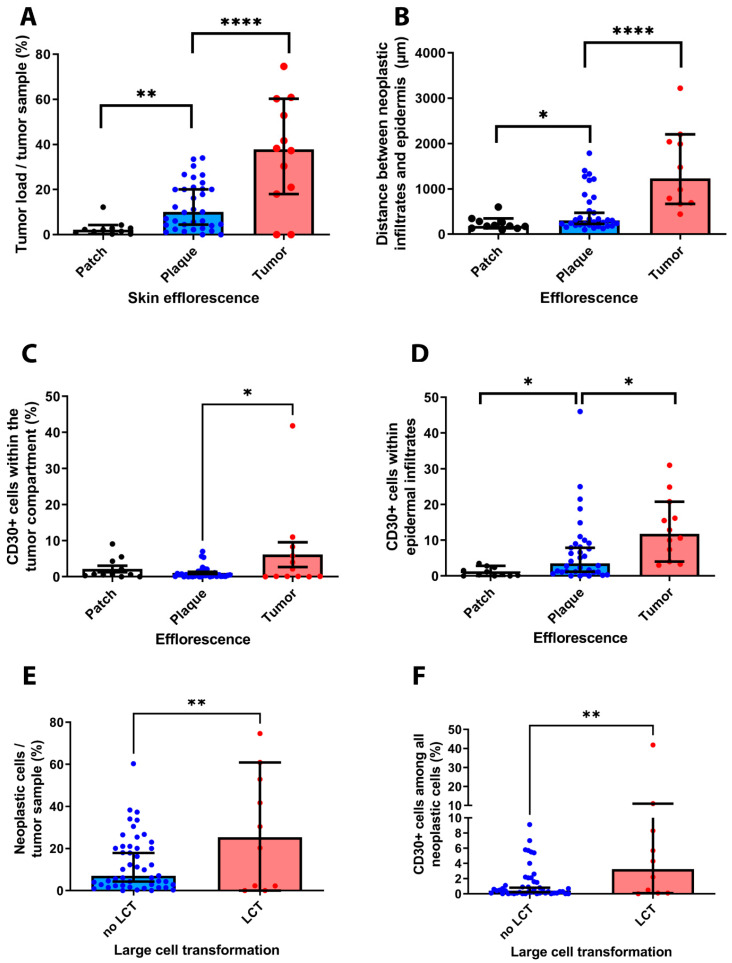
Correlation of results obtained from IHC analysis and clinical-pathological features of the investigated patient cohort. Our results revealed that the clinical stage of MF is correlated with the overall number of neoplastic cells within each tissue sample (**A**). Furthermore, we could show that the distribution of immune cell infiltrates correlated with the MF stage (**B**). Notably, the percentage of CD30+ T cells was significantly higher in tissue samples excided from advanced MF stage patients as compared to early MF stages (**C**). In these advanced MF stages, we could also observe a stronger infiltration of CD30+ T cells in the epidermis (**D**). Last, our results indicate a close association of the presence of a large-cell transformation, the overall burden of neoplastic cells and the occurrence of CD30+ cells (**E**,**F**). * p < 0.05, ** p < 0.005, **** *p* < 0.0001.

Next, we investigated whether our results obtained from immunohistochemistry might correlate with the clinical features of the examined patient cohort. In order to correlate the different stages of MF with results from IHC, we have carried out multiple *t*-tests. First, we have found that the proportion of T cells was significantly higher in tissue samples excided at the advanced MF tumor stage (*r* = 0.719, *p* < 0.001). Moreover, we could reveal a significant correlation between the amount of CD30+ T cells within the tumor infiltrates and the different MF stages (*r* = 0.773, *p* = 0.016). Despite an overall smaller infiltration of cells into epidermal structures found in tumor samples, we could observe that the proportion of CD30+ cells infiltrating the epidermis was significantly higher in samples excided at the MF tumor stage (*r* = 0.239, *p* = 0.013) (see Figure 6A–D). However, there was no association between the different tumor stages and the expression of other immune cell markers in the examined tissue samples (CD4, *p* = 0.62; CD8, *p* = 0.31; CD20, *p* = 0.88).

With regard to the spatial distribution of lymphocytes, we could demonstrate that the calculated median distance of lymphocyte infiltrates to epidermal structures significantly increased for patients´ samples, which have been excided at the MF tumor stage, as compared to early MF lesions (*r* = 0.494, *p* = 0.003) as the cells penetrated deeper into the dermis. 

Our analysis further unveiled a strong association between the overall tumor burden, the occurrence of LCT and the presence of CD30 positive cells within the examined tissue sections (see Figure 6E,F). In particular, we observed a significant association of the MF stages with the occurrence of LCT in the investigated tissue samples (*r* = 0.373, *p* = 0.027) (not shown). Furthermore, our results suggest a strong correlation between the amount of cells in each tumor tissue and the presence of LCT (*r* = 0.455, *p* = 0.007). Last, our analysis showed that the occurrence of LCT was also significantly associated with a stronger infiltration of tumor samples by CD30+ cells (*r* = 0.54, *p* = 0.001). 

### 3.4. MF Immune Phenotype at Different Stages of Tumor Progression

**Figure 7:**
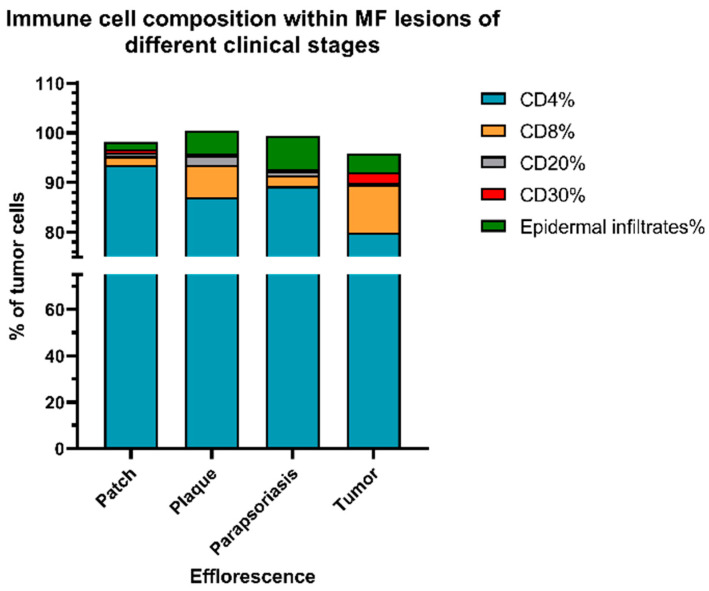
Immune cell composition at different stages of MF. Results show that the percentage of CD8+ T cells and CD30+ lymphocytes within the lymphocyte infiltrate substantially increased with a higher MF stage. Meanwhile, epidermal infiltration of cells was most dominant in the plaque and parapsoriasis lesions of MF. Notably, the infiltration by reactive B-cells was weak in all stages of MF.

**Figure 8:**
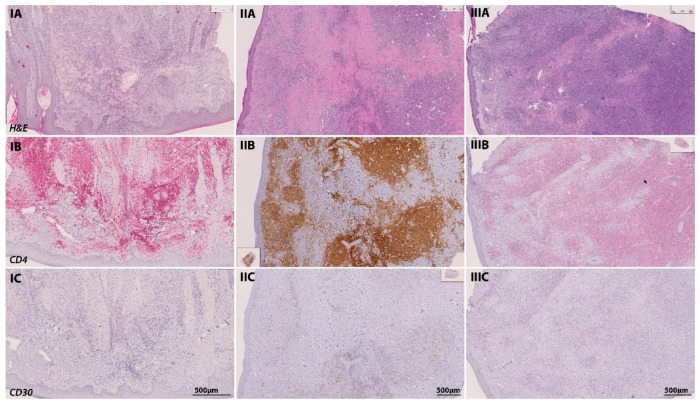
Temporal transformation of the MF immune phenotype of a 65-year-old patient in the course of tumor progression. At initial diagnosis in June 2016, the patient presented with a plaque-like lesion (**IA**–**IC**), which revealed a strong infiltration of CD4-positive cells in IHC (stained with PermaRed, red) (**IB**). Histological examination showed no signs of LCT or CD30-positive cells. In February 2019, the patient progressed into the tumor stage. In subsequent tumor biopsies (**IIA**–**IIC**), we could observe a transformation of the infiltrating lymphocytes with signs of LCT and a stronger CD30 positivity among CD4 positive lymphocytes in IHC (stained with DAB, brown). Another follow-up excision from a tumor lesion was performed in May 2019 (**IIIA**–**IIIC**) after the initiation of chemotherapy with CHOP and radiation therapy. Again, immunohistochemistry revealed a strong folliculotropism with the co-occurrence of LCT and a strong CD30 positivity (stained with PermaRed, red) in the infiltrates.

Next to the interindividual heterogeneity of MF, we also sought to assess the intraindividual heterogeneity of the MF immune phenotype in the course of tumor progression—also termed as temporal heterogeneity. To this end, we analyzed the tissue specimens from patients for whom two or more tissue samples were available and which had been obtained at different time points and at different clinical stages of the diseases. Among the 35 patients, 15 patients and 38 tissue samples have been eligible for this analysis. In order to assess the phenotypical changes between the different MF stages, we quantified the immune cell composition within the MF lesions by the presence of the different immune cell subtypes. Here, we could show that the dominant immune cell subtype in early-stage MF T cell infiltrates were CD4+ helper T-cells, whereas CD8+ cytotoxic T-cells and CD30+ cells were found to a much lesser degree. By contrast, analysis of tumor samples from these same patients at a later time point unveiled a stronger expression of CD8 and CD30 (Figure 7 and Figure 8). In this time-dependent analysis, we could also confirm the observations from Section 3.2. (see above) that LCT only occurred with the progression to the tumor stage, which was accompanied in most cases by a stronger expression of CD30 in cells (*r* = 0.437, *p* < 0.001). 

### 3.5. Prognostic Significance of the Investigated Markers

**Figure 9:**
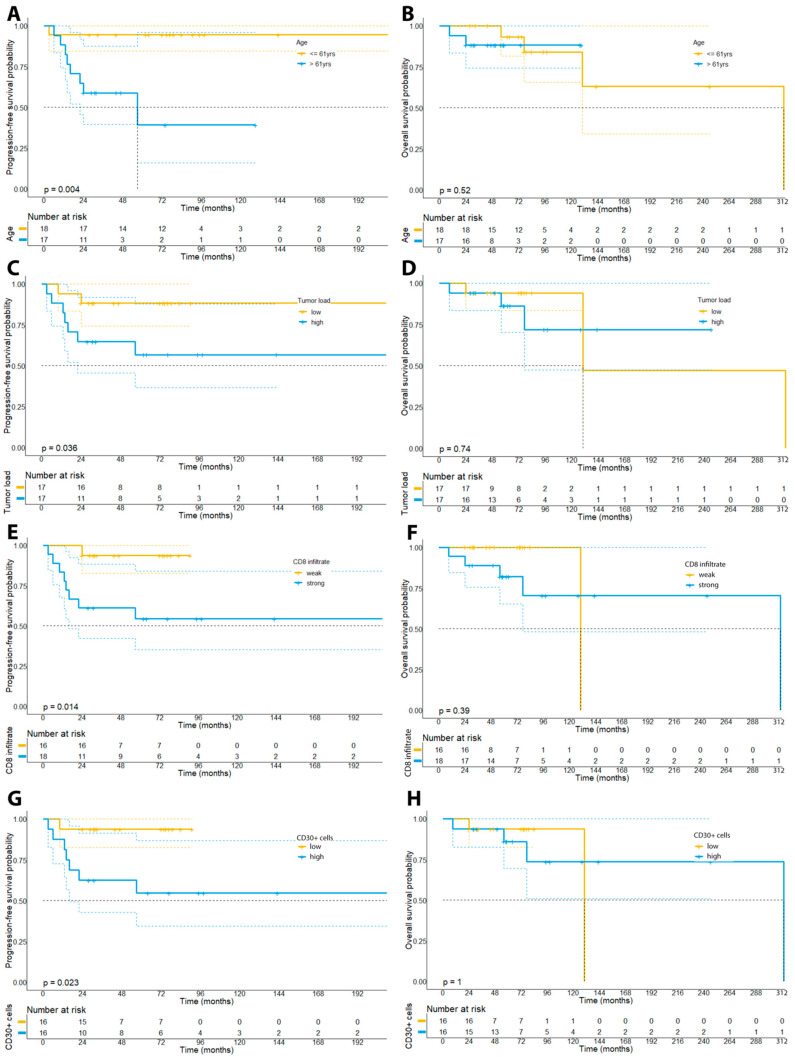
Kaplan–Meier plots depicting the survival curves for PFS and OS of the patient cohort dichotomized by age (median: 61 years; panels (**A**,**B**)), the tumor burden (median: 18.9%, panel (**C**,**D**)), the percentage of CD8+ lymphocytes in the examined tissue samples (median: 27.8%, panel (**E**,**F**)), and the percentage of CD30+ cells (median: 7.8%, panel (**G**,**H**)).

Last, we investigated the prognostic significance of the investigated clinical and IHC markers. After dichotomization of the patient cohort based on the median of the percentage of neoplastic cells (collectively termed as “tumor load” in the following: median: 18.9%), we could find a trend towards a shorter PFS (91 vs. 215 months, log rank *p* = 0.11) and OS (130 months vs. not reached, log rank *p* = 0.74) for patients showing a higher tumor load, albeit this correlation was below statistical significance (Figure 9C,D). 

**Figure 10:**
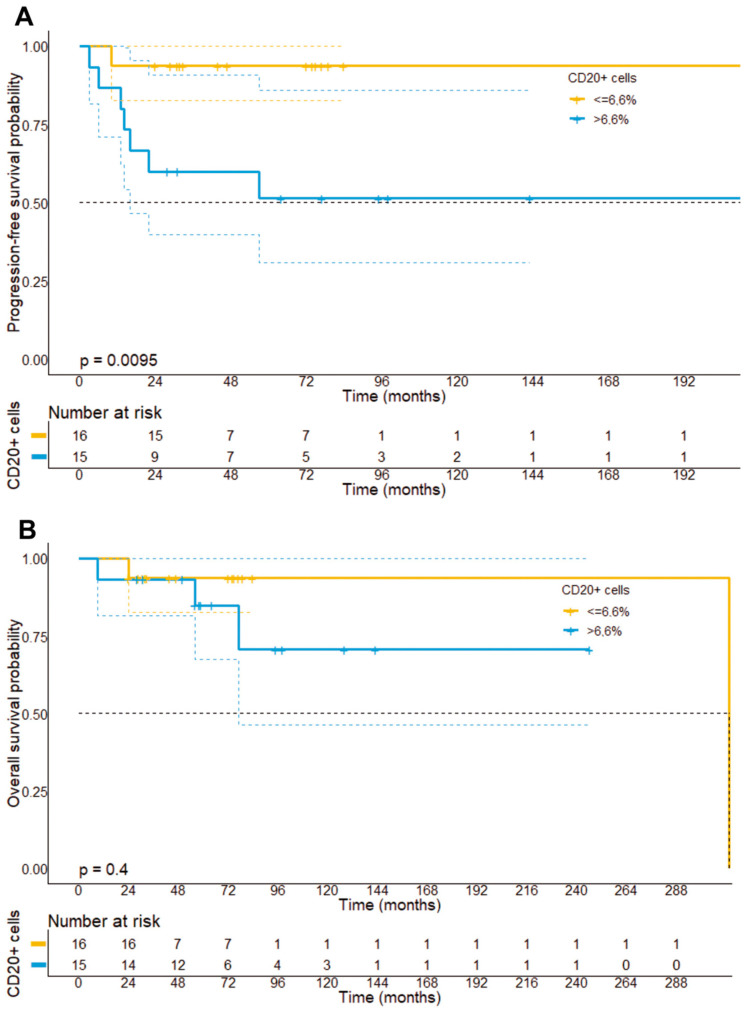
Progression-free survival (**A**) and overall survival stratified (**B**) by the median infiltration of CTCL by CD20+ B cells. It can be found that patients with a strong infiltration of CD20+ B cells had a significantly shorter PFS (*p* = 0.0095), but not OS as compared to patients with a small infiltration by CD20+ B cells.

In accordance with previous observations, we could not find a prognostic impact of the number of CD4+ lymphocytes neither on PFS (223 vs. 215 months, *p* = 0.24) nor OS (313 months vs. not reached, *p* = 0.43). In particular, we have dichotomized patients based on the median percentage of CD4+ lymphocytes (80.6%) within the whole-slide tissue samples. Contrary to our expectations, our analysis did, however, reveal that patients with a stronger infiltration by CD8+ lymphocytes within the tumor cell compartment had a higher risk of disease progression (*p* = 0.031), showed a shorter PFS (215 months vs. not reached, *p* = 0.038), but not OS (130 vs. 313 months, *p* = 0.25) (Figure 9E,F). Moreover, our analysis unveiled that a stronger infiltration by CD20+ B-cells based on the median percentage of CD20+ cells within the tissue samples (median: 6.6%) was associated with a higher risk of disease progression (*p* = 0.0023), a shorter PFS (120.4 months vs. 210 months, 95% CI: 174–245.4 months vs. 65.9–175 months, *p* = 0.009), but not OS (313 months vs. not reached, *p* = 0.40) (Figure 10). 

Aligning with previous reports, we could further observe a significant association of the percentage of CD30+ cells (median: 7.8%) with the risk of disease progression (*p* = 0.023) and PFS (not reached vs. 215 months, *p* = 0.023), but not with OS (not reached vs. 130 months, *p* = 0.30) (Figure 9G,H). Notably, we could not find a significant correlation of a LCT with the prognosis of patients (*p* = 0.264). 

Next to the baseline number of T-cell subsets or CD30+ cells, recent studies demonstrated that the spatial organization and the functional immune state of the tumor microenvironment might rather be of prognostic relevance [1]. Therefore, we have investigated whether the spatial distribution of immune cells and epidermal cells might be of prognostic relevance. However, our analysis revealed that the distance of tumor-infiltrates to epidermal structures was not prognostic (*p* = 0.55) (Supplementary Figure S1). 

With regard to clinical features of our patient cohort, we could observe that older patients (>61 years) showed a higher risk of disease progression (*p* = 0.015) and a shorter PFS (215 months vs. not reached, *p* = 0.032), although older patients did not show a significantly shorter OS (*p* = 0.86). 

## 4. Discussion

Histologic diagnosis and individual patient prognosis of early MF are challenging because of the substantial overlap between clinicopathological features of MF and various inflammatory skin conditions [5,20] and a definitive diagnosis often requires a combination of histological, immunohistochemical and molecular tests as well as clinicopathological correlation. IHC analysis typically allows for better characterization of the immune phenotype of MF, the aberrant expression of T cell antigens, and the spatial organization of T cells within the MF lesions. In the present whole-slide IHC study using digital histology with automated segmentation and artificial intelligence for cell characterization, we investigated the clinical and immunopathological features of a cohort of 35 MF patients and analyzed the expression of immune cell markers within the lymphocyte compartment in 58 available MF tissue samples. 

In accordance with previous reports, our results confirmed that LCT in MF was associated with an aggressive clinical course and poor survival, albeit this association was below statistical significance in our small patient cohort [10,11,21]. Notwithstanding, in our present study, we could observe an indolent course of MF for 2 to almost 20 years in 3 of 7 patients with a LCT in tissue samples. These observations underscored the need to define other parameters that may help predict which patients will be more likely to follow an aggressive rather than an indolent course and show adverse prognostic effects. Since previous studies on this issue showed rather conflicting data, we analyzed both the clinical and immunophenotypical features of our patient collective [22]. 

Overall, the median survival of the 35 patients was 246 months (range 9–313) and the 5-year progression-free survival was 72.6%. Our results confirmed previous studies that have shown that the proportion of CD30+ correlated with disease stage and exerted an adverse prognostic role [18]. Previous studies further indicated that the expression of CD30 might be associated with the histologic transformation of MF. In this study, we could show that the expression of CD30 did indeed show an increase with clinical stage and was further associated with a higher tumor burden and the occurrence of large-cell transformation. However, it remains unclear which transformative event might cause the sudden increase in CD30 expression or whether a strong CD30 expression coincides with the occurrence of large-cell transformation within the lymphocyte infiltrate. In contrast to a previously published series in patients with transformed MF, we could not find a significant association between folliculotropic MF and reduced survival of risk of disease progression. [2,23]. 

Older age is another documented prognostic indicator, which has been supported by our data using a cut-off of 61 years [18]. In contrast to previous observations, our data further indicate that a higher degree of CD8+ T lymphocytes within the tumor cell compartment exerted an adverse prognostic effect [7,24,25]. However, the difficulty of distinguishing between neoplastic T cells and the reactive T cell infiltrate might complicate inter-study comparisons. For this determination, single-cell studies by next-generation sequencing, for example, are needed. These methods call for fresh samples that could not be gathered in this study. On the other hand, we observed that a stronger infiltration by CD20+ immune cells also exerted an adverse prognostic effect, which is in accordance with previous studies that found a clinical regression of MF upon local injection with anti-CD20 antibodies [26]. Although the functional role of tumor-associated B cells in the TME of MF has not been fully clarified, our results, as well as other studies by Nielsen et al., indicate that B-cells might exert a clinically relevant role in MF progression and prognostication [27]. 

In addition to the analysis of clinical and histological prognostic parameters, this whole-slide IHC study is, to our knowledge, the first to describe the temporal changes within the MF tumor microenvironment for an extended follow-up period. Our results revealed that the MF tumor microenvironment is highly dynamic in the course of tumor progression and that temporal transformation particularly includes a large-cell transformation, a stronger expression of CD30 within these lymphocytes and a stronger infiltration of MF lesions by CD8+ T lymphocytes. This observation is in contrast to previous findings, which revealed that the T cell immunophenotype in the peripheral blood was stable and reproducible over time [3]. 

Despite the premature nature of our spatial analysis, we could further show that identification of MF disease stages can be facilitated by using the spatial distance between the cell infiltrate and the epidermis. For future analysis, it will also be highly interesting to further dissect the spatial distribution of different lymphocyte subsets within the MF lesions in order to identify potential mechanisms of malignant transformation and rapid disease progression in the organizational pattern of MF. 

Our study has several limitations that should be considered when interpreting the results from our investigations. First, as a tissue-based method, our IHC analysis was restricted to the T cell immunophenotype of MF lesions in the skin, whereas for a comprehensive analysis of the MF T-cell immunophenotype, an additional analysis of blood samples and other lymphatic tissues would have been required. Further, it has been reported that IHC analysis of skin samples shows several limitations compared to flow cytometric analysis of blood or MF skin lesions, such as an underrepresentation of CD8+ MF lesions and an overrepresentation of CD7 deletions [3]. Second, future studies with multiparameter histologic examinations are needed to better characterize the heterogeneity of T-cell subsets and their functional state across patient groups and better discriminate between lymphocytes and reactive lymphocyte subsets. Another limitation of our study is that tissue samples were collected at various time points in the course of disease progression, impairing our ability to directly compare the MF immune phenotype between different patients. Last, larger prospective studies will be needed in order to validate our findings in a bigger patient cohort and also take into account different pretreatment regimens that might have affected the MF immune phenotype. 

In summary, we could confirm the prognostic relevance of large-cell transformation in MF and its strong association with the presence of CD30+ lymphocytes. In contrast to previous reports, our data suggest an adverse prognostic role for CD8+ T cells in MF patients. Despite the small number of patients investigated, our results further indicate that the MF immune phenotype within the TME shows a strong temporal heterogeneity and is altered in the course of tumor progression. 

## Figures and Tables

**Table 1:** Antibodies (AB), immunohistochemistry (IHC) protocol and resulting staining patterns.

Antigen	Primary Antibody						Staining Pattern
	Cat.-No.	Clone	Primary AB Supplier	Species	Dilution	Incubation	
CD3	NCL-L-CD3-565	LN10	Novocastra	IgG1	1:200	60 min at room temperature	Membranous
CD4	104R-26	EP204	Cell Marque	IgG	1:25–1:100	60 min at room temperature	Membranous
CD8	M7103	C8/144B	Dako	IgG1	1:50–1:100	60 min at room temperature	Membranous
CD20	120M-85	L26	Cell Marque	IgG2a	1:100–1:500	60 min at room temperature	Membranous
CD30	130M-94	Ber-H2	Cell Marque	IgG1	1:50–1:200	60 min at room temperature	Membranous

## Supplementary Materials

The following supporting information can be downloaded at: https://www.mdpi.com/article/10.3390/cells11223570/s1, Figure S1: Progression-free and overall survival stratified by the median distance from CTCL infiltrates to epidermis.

## Abbreviations

CI	Confidence interval
CTCL	Cutaneous T cell lymphoma
ICI	Immune checkpoint inhibitors
H&E	Haematoxylin and eosin
IHC	Immunohistochemistry
LCT	Large-cell transformation
mAb	Monoclonal Antibody
MF	Mycosis fungoides
OS	Overall survival
PFS	Progression-free survival
ROI	Region of interest
TP	Tumor progression
TME	Tumor microenvironment
