# Supplementary material for: The Role of the Immune Phenotype in Tumor Progression and Prognosis of Patients with Mycosis Fungoides: A Quantitative Immunohistology Whole Slide Approach
Source: Cells. 2022 Nov 11;11(22):3570. doi: 10.3390/cells11223570 (PMC9688439; doi:10.3390/cells11223570)
Supplement: Supplementary file 1 [file cells-11-03570-s001.zip › cells-1851856-supplementary.pdf]

Supplementary Material

The Role of the Immune Phenotype in Tumor Progression and Prognosis of Patients with Mycosis Fungoides: A Quantitative Immunohistology Whole Slide Approach

Natallia Aulasevich [1], Maximilian Haist [1], Sebastian Försch [2], Beate Weidenthaler-Barth [1] and Volker Mailänder [1,3,*]

[1] Department of Dermatology, University Medical Center of the Johannes Gutenberg University Mainz, 55131 Mainz, Germany
[2] Institute of Pathology, University Medical Center of the Johannes Gutenberg University Mainz, 55131 Mainz, Germany
[3] Max Planck Institute for Polymer Research, 55128 Mainz, Germany
* Correspondence: mailaend@uni-mainz.de

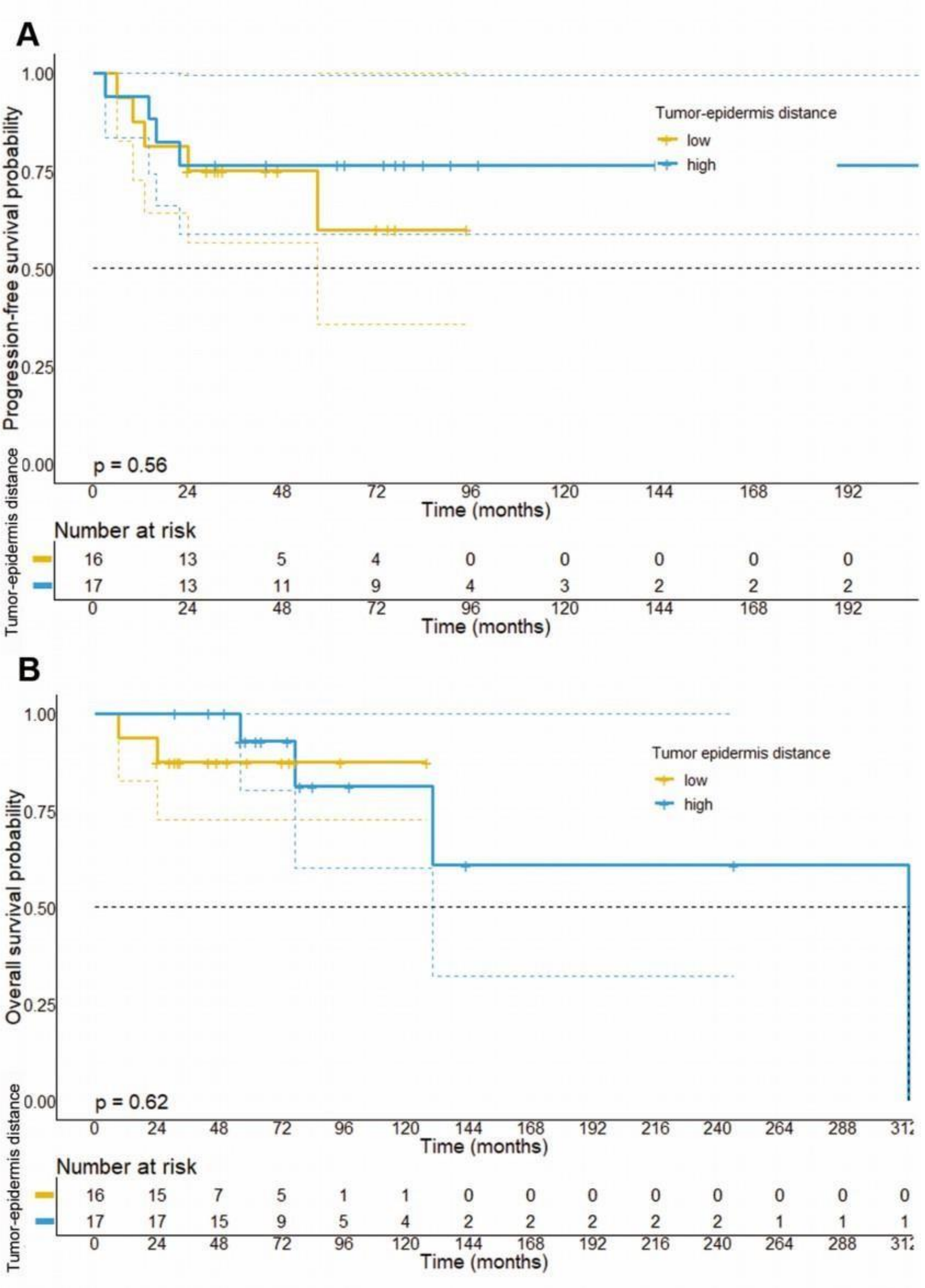

Supplementary Figure S1: Progression-free and overall survival stratified by the median distance from CTCL infiltrates to epidermis. We observed no significant differences in the OS and PFS of patients with small distances from CTCL infiltrates to epidermal cells (low) as compared to patients with big distances.
